# Expression of VEGF-A, Otx Homeobox and p53 Family Genes in Proliferative Vitreoretinopathy

**DOI:** 10.1155/2013/857380

**Published:** 2013-10-21

**Authors:** Claudio Azzolini, Ilaria Stefania Pagani, Cristina Pirrone, Davide Borroni, Simone Donati, Muna Al Oum, Diana Pigni, Anna Maria Chiaravalli, Riccardo Vinciguerra, Francesca Simonelli, Giovanni Porta

**Affiliations:** ^1^Department of Surgical and Morphological Sciences, Section of Ophthalmology, University of Insubria, Ospedale di Circolo, Via F. Guicciardini 9, 21100 Varese, Italy; ^2^Department of Clinical and Experimental Medicine, University of Insubria, Via O. Rossi 9, 21100 Varese, Italy; ^3^Ph.D. Program in Biotechnology, School of Biological and Medical Sciences, University of Insubria, Via O. Rossi 9, 21100 Varese, Italy; ^4^Pathology Institute, Ospedale di Circolo, Via F. Guicciardini 9, 21100 Varese, Italy; ^5^Eye Clinic, Second University of Napoli, Via S. Pansini 5, 80131 Napoli, Italy

## Abstract

*Introduction*. Proliferative vitreoretinopathy (PVR) is a severe inflammatory complication of retinal detachment. Pathological epiretinal membranes grow on the retina surface leading to contraction, and surgery fails in 5% to 10% of the cases. We evaluated the expression of VEGF-A, Otx1, Otx2, Otx3, and p53 family members from PVR specimens to correlate their role in inducing or preventing the pathology. *Methods*. Twelve retinal samples were taken from patients affected by PVR during therapeutic retinectomies in vitreoretinal surgery. Gene expression was evaluated using quantitative real-time reverse transcriptase PCR analysis and immunohistochemistry, using four healthy human retinae as control. *Result*. Controls showed basal expression of all genes. PVR samples showed little or no expression of Otx1 and variable expression of VEGF-A, Otx2, Otx3, p53, and p63 genes. Significant correlation was found among VEGF-A, Otx2, p53, and p63 and between Otx1 and Otx3. *Conclusions*. Otx homeobox, p53 family, and VEGF-A genes are expressed in PVR human retina. We individuated two possible pathways (VEGF-A, Otx2, p53, p63 and Otx1 and Otx3) involved in PVR progression that could influence in different manners the course of the pathology. Individuating the genetic pathways of PVR represents a novel approach to PVR therapies.

## 1. Introduction 

Proliferative vitreoretinopathy (PVR) is a complication of a retinal detachment and occurs in approximately 8–10% of patients developing retinal detachment [1–3]. In retinal detachment, a full-thickness retinal break exposes cells, allowing macrophages, retinal pigment epithelial cells, glial cells, and fibroblasts to migrate into the vitreous, a rich source of growth factors and cytokines correlated to PVR activity [4–6]. All these cells proliferate in the vitreous, survive, form extracellular matrix proteins, and assemble into membranes. These membranes contract on the retina, causing PVR and subtending chronic inflammation [7]. In the anterior segment of the eye, elevated laser flare photometry value in aqueous humor corresponds to an altered profibrotic intraocular cytokines milieu [8].

PVR can be divided into multiple categories based on the configuration of the retina and the location of the scar tissue [6, 9], with surgery as unique therapeutic option [10]. Despite advances in surgical techniques, the percentage of unhealed PVR remains high, causing from 5% to 10% failures in retinal surgical repairs. For the above reasons, in the recent years it has become increasingly important to individuate the inflammatory and genetic mechanisms involved in the pathogenesis of PVR, so as to highlight a possible design to be exploited in clinical trials [11–16]. 

Vascular endothelial growth factor A (VEGF-A), platelet-derived growth factors (PDGFs), and non-PDGFs (growth factors outside of the PDGF family) are relevant to PVR pathogenesis because of their role in suppressing p53 levels through different pathways [17]. This promotes an environment of cell survival, proliferation, organization into a membrane, and subsequent membrane contraction, all processes relevant and intrinsic to PVR pathogenesis. 

The dynamic regulation of VEGF by the p53 family members makes its regulation complex, especially given the fact that all of the three transcription factors (p53, p63, and p73) are able to induce and repress VEGF, which appears to be dependent on cellular context and stimulus [18]. The OTX family includes an important class of Homeodomain-containing transcription factors involved in the induction and in the morphogenesis of the neuroectoderm, leading to the formation of the vertebrate central nervous system that includes the retina. The Otx1 gene is expressed in the rostral part of the neural tube and is required for corticogenesis and sense organs development. Postnatally, it is a marker of the anterior part of the retina, which then will develop into the ciliary body. Otx2 plays a role in the functional development of the retina, in which it is expressed at both prenatal and adult stage; it is necessary for the development and differentiation of rod and cone photoreceptors and bipolar cells, and it is detected in the retinal pigment epithelium. In addition, Otx3 expression was observed in the eye development during embryogenesis [19].

The aim of the present study is to evaluate the expression levels of VEGF-A, Otx1, Otx2, Otx3, and p53 family genes in adult healthy human retinae in comparison with retinae affected by PVR and to try to understand their role in inducing or preventing this eye pathology.

## 2. Materials and Methods 

### 2.1. Retina Samples

Twelve human retinae samples were taken from twelve patients affected by PVR during vitreoretinal surgery by the same operating surgeon (CA). PVR was graded according to the Retina Society Terminology Committee [9]. Patients' data are shown in Table 1. All patients were instructed and they signed the informed consent (Ospedale di Circolo, Varese, Italy). Four adult human healthy retinae from autopsy were used as control tissue. The research was approved by the local ethical committee and followed the tenets of the Declaration of Helsinki.

### 2.2. Surgery

**Figure 1:**
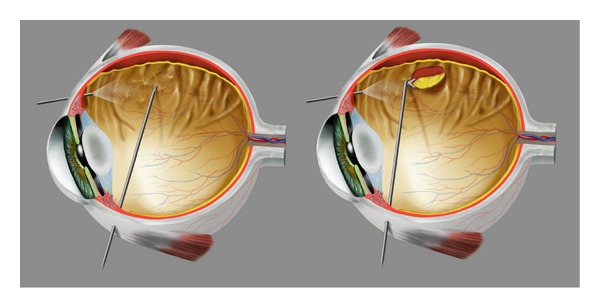
Important surgical steps. Left: peeling of epiretinal membranes using endoillumination and surgical instruments entering the eye through sclerotomies. Right: cut of peripheral retina (retinectomy) allowing further retinal reattachment.

**Figure 2:**
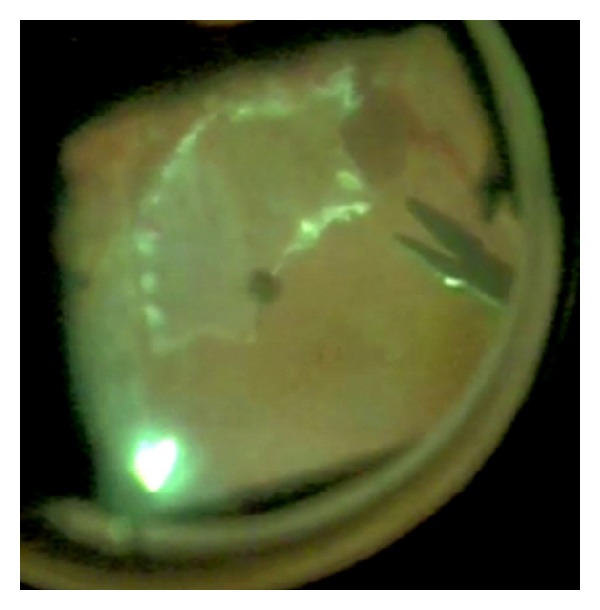
Retina grasp. After retinectomy, a small retinal portion is grasped with nontraumatic forceps and is removed from the eye.

Surgical procedures were similarly conducted in all patients. After accurate vitreous and vitreous base removal with vitrectomy (Stellaris, Bausch&Lomb, Rochester, New York, USA), endoilluminator and room temperature infusion fluid at surgical microscope (OPMI 1, Carl Zeiss, Jena, Germany), and accurate epiretinal membranes' peeling with surgical instruments, it was impossible to reattach the peripheral retina because of strong epi- and intraretinal PVR tissue in peripheral retina. Therefore, peripheral retinectomy preceded by endodiathermy of adjacent retina tissue was necessary in each patient to allow final total retinal reattachment at the end of surgery (Figure 1). Retina removed from retinectomy is usually destroyed for preventing other PVR occurrences and for avoiding anterior neovascularization of the eye stimulated from VEGF coming from the ischemic retinal portion. In these cases, the little retinal specimens were isolated, grasped with nontraumatic instruments (Figure 2), and removed via pars plana through the sclerotomy holes used for entering surgical instruments. The retina sample was placed in RNA later solution (Ambion, Austin, TX, USA) and stored at −20°C until RNA extraction. Surgery continued with the use of perfluorocarbon liquid [20] which allows subsequent drainage of subretinal fluid by a Charles flut-needle and appropriate laser endophotocoagulation [1, 21]. Perfluorocarbon liquid-silicone oil 1000 centistokes exchange was later performed to have the final postoperative stable retina reattachment. Silicone oil was surgically removed after two to three months to reach stable retina reattachment and final visual acuity.

### 2.3. RNA Isolation and Reverse-Transcription

RNA was isolated using EuroGold Total RNA Mini Kit (Euroclone, Milan, Italy) and quantification of total RNA was performed by NanoDrop ND 1000 spectrophotometer (Thermo Scientific, Wilmington, USA). Total RNA (1 *μ*g) was reverse transcribed using High Capacity cDNA Kit (Applied Biosystems, Foster City, CA, USA) according to protocol.

### 2.4. Quantitative Real-Time Reverse Transcriptase PCR

Quantitative real-time reverse transcriptase PCR (qRT-PCR) was performed by TaqMan technology using ABI Prism 7000 apparatus (Applied Biosystems, Foster City, CA, USA). Gene expression analyses were done with TaqMan Assays-on-Demand containing primers and fluorescent probe mix (Applied Biosystems, Foster City, CA, USA). PCR reaction mix contained 12.5 *μ*L of TaqMan Universal PCR Master Mix, no AmpErase UNG (Applied Biosystems, Foster City, CA, USA), 1.25 *μ*L Assays-on-Demand, 3 *μ*L of cDNA, and 8.25 *μ*L of nuclease-free water. Thermocycler program consists of an initial hot start cycle at 50°C for 2 minutes and 90°C for 10 minutes, followed by 40 cycles at 95°C for 15 seconds and a final cycle at 60°C for 1 minute. For all genes, reactions were performed in triplicate. Negative control consists of PCR mix without cDNA. Human beta-actin gene was used as endogenous control to normalize gene expression levels for relative quantitative analysis through comparative cycle threshold (ΔCt) method. Finally, ΔΔCt method was used to compare gene expressions between human adult healthy retina and human retinal tissues from PVR patients.

### 2.5. Statistical Analysis

Statistical correlations between gene expressions were calculated with dispersion plots. We correlated two genes for graphic and values were considered significant with a linear regression coefficient (*R*) > 0.8 [22].

### 2.6. Immunohistochemistry

Immunohistochemical analyses were performed on formalin-fixed, paraffin-embedded sample of healthy human retina used as control. Three *μ*m sections were mounted on poly-L-lysine-coated slides, deparaffinized and hydrated through graded alcohols to water. Endogenous peroxidase activity was blocked with 3% aqueous hydrogen peroxide for 10 minutes. Antigen retrieval was performed with citrate buffer (10 mM, pH 6.0) inside a 720W domestic microwave oven. The section was incubated overnight at 4°C with rabbit anti-Otx2 polyclonal antibody (Chemicon International, Temecula, CA, cat. no. AB9566) at a dilution of 1 : 2000 and later with Ultravision Detection System Kit (Thermo Scientific, Fremont, CA) according to the manufacture's suggestion. The immunoreaction was developed using 0.03% 3.3′-diaminobenzidine tetrahydrochloride (Sigma-Aldrich, St. Louis, MO, USA), and nuclei were counterstained with Harris hematoxylin. The primary antibody was produced using full-length recombinant human Otx2 as immunogen. Due to amino acid sequence homology between the Otx1 and the Otx2 proteins, the antibody recognized both of the proteins. Negative specificity control was performed by omission of primary antibody and substitution with a nonimmune serum with the same dilution.

## 3. Results 

### 3.1. Gene Expression Analysis in Adult Healthy Human Retina and in PVR Patients

**Figure 3:**
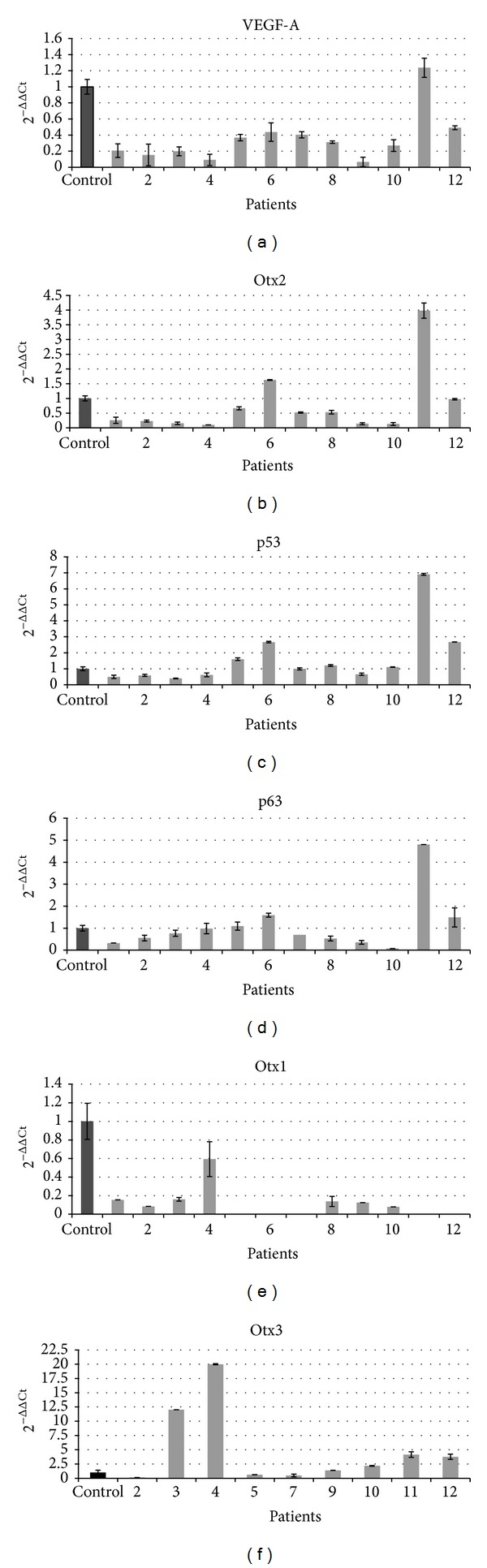
Gene expression levels in adult healthy human samples and in patients affected by PVR. Gene levels were detected by quantitative PCR. 2^−ΔΔCt^ values representing gene expression are shown on *y*-axis. Black column: adult human samples as controls; gray columns: PVR affected patients. VEGF-A, Otx2, p53, and p63 show the similar expression trend, inversely to Otx1 and Otx3.

**Figure 4:**
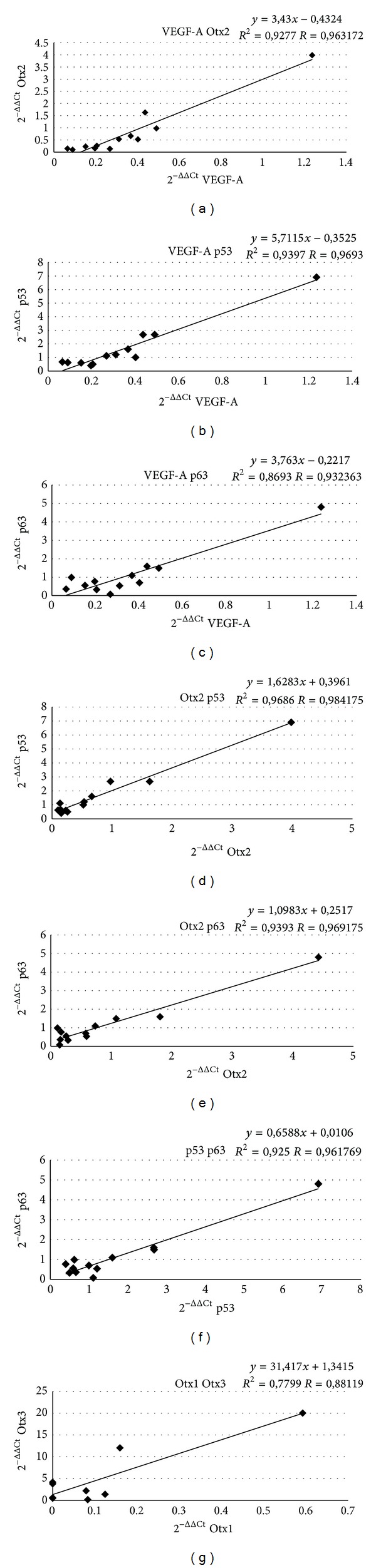
Statistical correlation among VEGF-A, Otx2, p53, and p63 genes and between Otx1 and Otx3 genes. Dispersion plots correlate two genes for graphic. *x*- and *y*-axes indicate 2^−ΔΔCt^ values that represent gene expression levels. Linear regression coefficient (*R*) > 0.80 indicates a statistically significant correlation.

To our knowledge, this is the first time that the expression of Otx genes is found in human adult retina and in PVR tissue. In the control samples, we found basal expression of all genes. In PVR samples, we found little (samples 1, 2, 3, 4, 8, 9, and 10) or no expression (samples 5, 6, 7, 11, and 12) of Otx1, attesting the nondifferentiated state of retinal tissues [23], and variable expressions of VEGF-A, Otx2, Otx3, p53, and p63. We found high Otx3 levels in samples with little levels of Otx2 (samples 3 and 4) (Figure 3). In particular, VEGF-A, Otx2, p53, and p63 genes showed the same expression trend (Figure 4). We found higher levels of VEGF-A, Otx2, p53, and p63 genes inversely to Otx1 and Otx3 expression in PVR samples affected by more severe features of the disease (more severe PVR, more number of surgical operations) (samples 5, 6, 7, 11, and 12). We could not evaluate Otx3 levels in patients 1, 6, and 8 due to the low amount of samples.

### 3.2. Statistical Analysis

qRT-PCR showed a correlation (*R* > 0.8) among VEGF-A versus Otx2, p53, and p63; Otx2 versus VEGF-A, p53, and p63; p53 versus VEGF-A, Otx2, and p63, and finally p63 versus VEGF-A, Otx2, and p53. Moreover we found a close correlation between Otx1 and Otx3. No statistical relationships were found between Otx1, Otx3, and all other genes (Figure 4).

### 3.3. Immunohistochemistry

**Figure 5:**
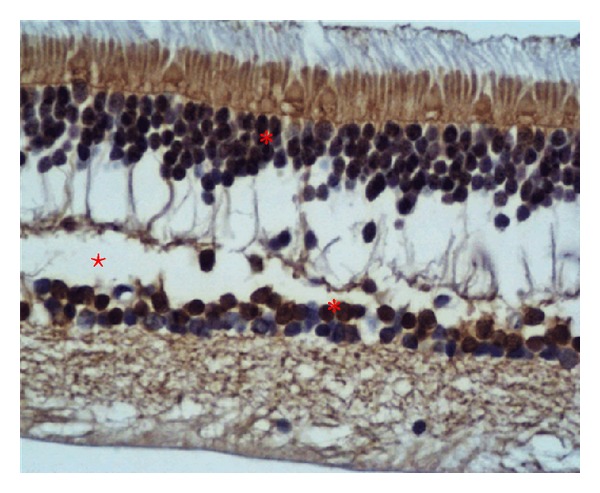
Histological section of healthy adult human retina. The different retinal layers appear marked in dark-marron with rabbit anti-Otx2 polyclonal antibody. Positivity is shown (from top to bottom) in photoreceptors, horizontal, bipolar, and neuronal cells (asterisks). An artifact (empty space) is present due to fixating procedure (star).

By immunohistochemical analysis we observed positivity for anti-Otx2 antibody in different retinal layers of adult healthy human sample. Photoreceptors (rods and cones), horizontal cells, bipolar and ganglion cells showed positivity for the Anti-Otx2 antibody, on the contrary of Muller cells. We confirmed the presence of Otx proteins in nucleus and cytoplasm of human retina (Figure 5).

## 4. Discussion

Zebrafish, like many members of the ray-finned fish (teleosts), have the innate capacity to regenerate tissues (e.g., fins, heart, and eye) [24]. In teleost fish, retinal neurogenesis continues in adult life beyond embryogenesis development. In addition, following the destruction of retinal neurons, the retina can regenerate and restore visual function [25]. Following injury, Muller glia cells dedifferentiate into a stem-like state and proliferate to replace lost retinal cells [24]. In the retina of the posthatch chick, the Muller glia demonstrates the ability to dedifferentiate into retinal progenitor, but significant regeneration of retinal neurons does not occur [26]. In contrast to the persistent neurogenesis in the retina of teleosts, neurogenesis in the retina of mammals is completed during pre- and perinatal development [27], and there is as yet no evidence for continual neurogenesis or regeneration in the adult retina. 

PVR is a proliferative disease and the knowledge of expressions of some key regulatory genes could be useful to understand the disease and, hopefully, retina regeneration. The purpose of the present study was to evaluate the expression of VEGF-A, Otx1, Otx2, Otx3, p53, and p63 genes in adult healthy human retina from autopsy and in surgically removed retinae of PVR affected patients.

We confirmed the presence of VEGF-A and p53 family members and, for the first time, we found the expression of Otx genes in controls, also confirmed by immunohistochemical analysis, and in PVR tissue (Figures 3 and 5). 

Statistical analysis showed a correlation among VEGF-A, Otx2, p53, and p63 and between Otx1 and Otx3 (Figure 4). These data could individuate two possible pathways involved in the pathogenesis of PVR. Molecular genetic pathways represent a hypothesis or model of how the expression of different genes in a series of biochemical relationships influences each other and eventually leads to a specific phenotypical expression [28]. 

The two groups of genes showed reverse trends. In fact, patients with higher expressions of VEGF-A, Otx2, p53, and p63 (samples 5, 6, 7, 11, and 12) showed no expression of Otx1 and low levels of Otx3 in comparison to the other samples. On the contrary, patients showing low levels of VEGF-A, Otx2, p53, and p63 genes (samples 3 and 4) had higher levels of Otx1 and Otx3. Thus, Otx1 and Otx3 showed a significant correlation, inversely to what is observed in Otx2 expression. In fact, it is reported that Otx3 significantly suppresses Otx2-induced transcription activity, suggesting that Otx3 functions as a transcription repressor of Otx2 by acting competitively on the consensus TAATCC sequence [15]. Moreover, patients who showed a very severe PVR and underwent an elevated number of surgical procedures (samples 5, 6, 7, 11, and 12) had higher levels of VEGF-A, Otx2, p53, and p63 genes. 

In our case series, the functionality of the retinae undergoing genetic study is similar at the time of surgery and at six months followup (results shown in Table 1): this evidence reflects the final outcomes of the literature, as expected, because we performed a standard surgery without new therapies. Aggressive surgery succeeded in stopping PVR development in most of the cases. The cases with more severe PVR and higher levels of VEGF-A, Otx2, p5, and p63 genes showed a worse final functional outcome.

It was demonstrated that p53 can influence VEGF-A expression both increasing and repressing its levels and that, likewise, the different isoforms of p63 have different effect on VEGF-A expression [17]. Further studies are needed to better understand the effective role and correlation among VEGF-A and p53 family members in retina and in retina affected by PVR.

During standard vitreoretinal surgeries using room temperature infusion fluid, the vitrectomy cavity and retina reach deep hypothermia. After closing the infusion line, intraocular tissue rewarms rapidly [29, 30]. These temperature fluctuations could influence many biological functions like bleeding, expression of cytokines in the vitreous, induction of specific patterns of proteins, and neuroprotection. Low temperature causes lower VEGF expression [31]. On the contrary, in our study we found higher VEGF gene expression, highlighting our results.

Immunohistochemical analysis showed the presence of Otx proteins in the internal part of photoreceptors, in horizontal cells, in bipolar cells, and in neuronal cells. Despite the fact that the role of Otx1, Otx2, and Otx3 has been proved during embryo development, little is known about their functions in adult human retina. We hypothesize that Otx2 could have a function of maintenance in the identity and survival of adult retinal differentiated cells, and that its high expression in PVR patients correlates with a reentry of differentiated cells into proliferation cycle and staminal status. Precursor cells, probably in an attempt to regenerate death retina cells, on the contrary could mature in fibroblasts that produce fibrocellular membranes. Interestingly, in very severe PVR cases this process is more stimulated and it is accompanied by higher expression of Otx2. 

One of the main targets of genetic studies is to translate evidence and benefits into clinical practice. In our study, we attempted to reach this objective in two different ways. Firstly, our study shows the elevated expression of VEGF-A in PVR patients, and VEGF-A was found to promote the bioactivity of vitreous in patients and rabbits with PVR. In the latter, the anti-VEGF ranibizumab injected intravitreally was found to neutralize PVR [17]. Therefore, in line with the results and the authors' opinion, it can be postulated that intravitreal ranibizumab could be effective in protecting patients from developing PVR. The intravitreal use of ranibizumab should be considered in the presence of PVR predisposing factors (i.e., retinal detachment secondary to trauma, long-lasting intraocular surgical procedures, or visible signs of PVR during standard surgery and during postsurgical followup). Secondly, during retinal detachment surgery, we could perform an intraoperative extemporaneous low-cost examination of retinal gene expression and, if high levels of PVR-related genes are shown, it could be useful to perform a more aggressive surgery instead of a low invasive one. Nowadays, technical and organizational hurdles do not allow performing this procedure in a useful timeframe; the exploitation of the above mentioned technique would be a valid attempt to try in the near future.

## 5. Conclusions

We found expressions of VEGF-A, p53 family, and, for the first time, Otx homeobox genes in healthy adult human retinae and in retinae affected by PVR. We individuated two possible pathways, VEGF-A, Otx2, p53, and p63 and Otx1 and Otx3 involved in PVR genes pattern that could influence in different manners the course of the pathology. In particular, in many samples of patients with more severe features of PVR, we found higher levels of VEGF-A, Otx2, p53, and p63 genes inversely to Otx1 and Otx3 expression. The anti-VEGF ranibizumab molecule, neutralizing retinal VEGF, could protect retina from PVR.

The immunohistochemical analysis of human healthy retina showed the presence of Otx proteins in many layers of the retina, confirming the hypothesis of their role in acting as a survival factor. In retinae with PVR, the highly expressed levels of Otx2 suggest the role of this gene in the proliferation of retinal stem cells as replacement for dead cells. Further studies are needed to better comprehend the genetic mechanism subtended to PVR exogenesis.

A better understanding of cellular and molecular mechanisms that regulate growth-associated induced neurogenesis in the retina may lead to new approaches for enhancing or controlling the proliferative and regenerative capacity for future therapeutic uses.

## Figures and Tables

**Table 1:** Patients' data in PVR surgery.

Patients	Age*	Sex	Refraction (SE)	Visual acuity before surgery*	PVR stage*	Total number of vitreoretinal surgeries*	Visual acuity final outcome**
1	65	F	−1.00	0.4	C2	2	0.4
2	58	M	−1.50	0.2	C2, CA1	2	0.3
3	67	M	−1.00	0.3	C3	2	0.2
4	62	M	+1.75	0.1	C6, CA2	5	0.1
5	62	M	+2.00	0.1	C8, CA3	6	0.05
6	45	M	−7.50	0.02	C7	2	HM
7	80	M	+1.00	HM	C6	4	HM
8	70	F	0	0.02	C5	2	HM
9	72	M	+1.00	0.2	C4	1	0.2
10	51	M	−1.00	HM	C6	4	0.05
11	77	M	0	HM	C7	2	HM
12	57	M	−6.00	HM	C11, CA4	2	LP

SE: spherical equivalent. HM: hand motion. LP: light perception. PVR: proliferative vitreoretinopathy. PVR stage: presence of preretinal or subretinal membranes posterior (C) or anterior (CA) to the equator of the eye, and number of clock hours involved (from 1 to 12). *at the time of retinectomy in PVR surgery. **six months after surgery.

## References

[1] Charles S, Calzada J (2010). *Vitreous Microsurgery*.

[2] Leiderman YI, Miller JW (2009). Proliferative vitreoretinopathy: pathobiology and therapeutic targets. *Seminars in Ophthalmology*.

[3] Ricker LJ, Kessels AGH, de Jager W, Hendrikse F, Kijlstra A, la Heij EC (2012). Prediction of proliferative vitreoretinopathy after retinal detachment surgery: potential of biomarker profiling. *The American Journal of Ophthalmology*.

[4] Pennock S, Rheaume M, Mukai S, Kazlauskas A (2011). A novel strategy to develop therapeutic approaches to prevent proliferative vitreoretinopathy. *The American Journal of Pathology*.

[5] Symeonidis C, Papakonstantinou E, Androudi S (2012). Interleukin-6 and matrix metalloproteinase expression in the subretinal fluid during proliferative vitreoretinopathy: correlation with extent, duration of RRD and PVR grade. *Cytokine*.

[6] Garweg JG, Tappeiner C, Halberstadt M (2013). Pathophysiology of proliferative vitreoretinopathy in retinal detachment. *Survey of Ophthalmology*.

[7] Moysidis SN, Thanos A, Vavvas DG (2012). Mechanisms of inflammation in proliferative vitreoretinopathy: from bench to bedside. *Mediators of Inflammation*.

[8] Hoerster R, Hermann MM, Rosentreter A, Muether PS, Kirchhof B, Fauser S (2013). Profibrotic cytochines in acqueous humor correlate with aqueous flare in patients with regmatogenous retinal detachment. *The British Journal of Ophthalmology*.

[9] Retina Society Terminology Committee Classification (1983). The classification of retinal detachment with proliferative vitreoretinopathy. *Ophthalmology*.

[10] Sadaka A, Giuliari GP (2012). Proliferative vitreoretinopathy: current and emerging treatments. *Clinical Ophthalmology*.

[11] Stryjewski TP, Vavvas DG (2013). Genetic correlates of proliferative vitreoretinopathy. *Investigative Ophthalmology and Visual Science*.

[12] Rojas J, Fernandez I, Pastor JC (2013). A genetic case-control study confirms the implication of SMAD7 and TNF locus in the development of proliferative vitreoretinopathy. *Investigative Ophthalmology and Visual Science*.

[13] Yu H, Li T, Zou X (2013). Effects of lysyl oxidase genetic variants on the susceptibility to rhegmatogenous retinal detachment and proliferative vitreoretinopathy. *Inflammation*.

[14] Asato R, Yoshida S, Ogura A (2013). Comparison of gene expression profile of epiretinal membranes obtained from eyes with proliferative vitreoretinopathy to that of secondary epiretinal membranes. *PLoS ONE*.

[15] Kazlauskas A (2003). Advances in a gene therapy-based approach to treat proliferative vitreoretinopathy. *Archivos de la Sociedad Espanola de Oftalmologia*.

[16] Pastor-Idoate S, Rodriguez-Hernández I, Rojas J (2013). The p53 codon 72 polymorphism (rs1042522) is associated with proliferative vitreoretinopathy: the retina 4 project. *Ophthalmology*.

[17] Pennock S, Kim D, Mukai S (2013). Ranibizumab is a potential prophylaxis for proliferative vitreoretinopathy, a nonangiogenic blinding disease. *The American Journal of Pathology*.

[18] Ghahremani MF, Goossens S, Haigh JJ (2013). The p53 family and VEGF regulation: ‘It's complicated’. *Cell Cycle*.

[19] Zhang Y, Miki T, Iwanaga T (2002). Identification, tissue expression, and functional characterization of Otx3, a novel member of the Otx family. *The Journal of Biological Chemistry*.

[20] Kertes PJ, Peyman GA (1997). Drainage of subretinal fluid under silicone oil. *Canadian Journal of Ophthalmology*.

[21] Azzolini C, Gobbi PG, Brancato R, Trabucchi G, Codenotti M (1996). New semiconductor laser for vitreoretinal surgery. *Lasers in Surgery and Medicine*.

[22] Ludbrook J (2008). Statistics in biomedical laboratory and clinical science: applications, issues and pitfalls. *Medical Principles and Practice*.

[23] Terrinoni A, Pagani IS, Zucchi I (2011). OTX1 expression in breast cancer is regulated by p53. *Oncogene*.

[24] White DT, Mumm JS (2013). The nitroreductase system of inducible targeted ablation facilitates cell-specific regenerative studies in zebrafish. *Methods*.

[25] Otteson DC, Hitchcock PF (2003). Stem cells in the teleost retina: persistent neurogenesis and injury-induced regeneration. *Vision Research*.

[26] Fischer AJ, Reh TA (2001). Müller glia are a potential source of neural regeneration in the postnatal chicken retina. *Nature Neuroscience*.

[27] Carter-Dawson LD, LaVail MM (1979). Rods and cones in the mouse retina. II. Autoradiographic analysis of cell generation using tritiated thymidine. *Journal of Comparative Neurology*.

[28] Waaijenborg S, Zwinderman AH (2009). Sparse canonical correlation analysis for identifying, connecting and completing gene-expression networks. *BMC Bioinformatics*.

[29] Romano MR, Garcia-Vallejo J, Vinciguerra P, Costagliola C (2013). Thermodynamics of vitreoretinal surgery. *Current Eye Research*.

[30] Landers MB, Watson JS, Ulrich JN, Quiroz-Mercado H (2012). Determination of retinal and vitreous temperature in vitrectomy. *Retina*.

[31] Klettner A, Faby H, Hillenkamp J, Roider J (2012). Temperature-dependent vascular endothelial growth factor (VEGF) induction in human retinal pigment epithelium—implications for transpupillary thermotherapy in uveal melanoma. *Acta Ophthalmologica*.

